# Diagnosis and Treatment of Pantothenate Kinase-Associated Neurodegeneration (PKAN): A Systematic Review

**DOI:** 10.7759/cureus.46135

**Published:** 2023-09-28

**Authors:** Meera R Pohane, Rajshri Dafre, Nikhil G Sontakke

**Affiliations:** 1 Medical Surgical Nursing, Shalinitai Meghe College of Nursing, Datta Meghe Institute of Higher Education and Research, Wardha, IND; 2 Health Sciences, Jawaharlal Nehru Medical College, Datta Meghe Institute of Higher Education and Research, Wardha, IND

**Keywords:** management, diagnosis of pkan, treatment, iron/lipofuscin in pantothenate kinase-related neurodegeneration, pantothenate kinase-associated neurodegeneration

## Abstract

A specific type of neurodegeneration with brain iron accumulation (NBIA) falls under the omit phenotypic continuum-early childhood development of progressive pantothenate kinase-associated neurodegeneration (PKAN). Classic PKAN is distinguished from atypical PKAN by stiffness, dystonia, dysarthria, and choreoathetosis. Pigmentary retinal degeneration is a widespread cause of classic PKAN. Atypical PKAN is distinguished by a later onset (>10 years), noticeable speech abnormalities, psychological disorders, and slower disease development. Studies designed to support various PKAN therapeutic strategies have highlighted the intricacy of coenzyme A (CoA) metabolism and the limitations of our present understanding of disease causation. Therefore, improvements in our knowledge of the causes and therapy of PKAN may have ramifications for our comprehension of other, more prevalent diseases. They may also shed fresh light on the physiological significance of CoA, a cofactor essential for the operation of several cellular metabolic processes. The existence of low but considerable PANK2 expression, which can be elevated in some mutations, provides necessary information that can justify using a hefty dose of pantothenate as a treatment. A more effective therapeutic approach can be achieved by comparing the effects of various currently available pharmacological alternatives on the pathophysiological alterations in fibroblasts and neuronal cells obtained from PKAN patients. The objective of this study is to educate and inform people about PKAN disease conditions such as treatment, diagnosis, and complications. These cell models will also help evaluate the effectiveness of future medicinal innovations. This review discusses the neurodegeneration generated by pantothenate kinase in cellular models, iron/lipofuscin in pantothenate kinase-related neurodegeneration, and treatment and diagnosis of PKAN.

## Introduction and background

Extrapyramidal movements and brain iron buildup issues are both symptoms of the uncommon autosomal-recessive syndrome pantothenate kinase-associated neurodegeneration (PKAN), which causes neurodegeneration [1]. PKAN is an inborn defect causing dystonia, parkinsonism, and iron buildup [2]. Substantia nigra and globus pallidus are affected by an autosomal recessive brain disorder brought on by PANK2 mutations [3]. Walking, speech, and writing issues were the most prevalent early symptoms or functional limits reported in patients (often not restricted to just one symptom), followed by a number of additional first signs or symptoms that are less common, like emotional and behavioral problems and dystonia [4]. There will be a discussion of unique copper, iron, and manganese storage disorders with neuropsychiatric symptoms. Clinicians can benefit from the current thinking as they make a differential diagnosis [5]. Due to mutations in the pantothenate kinase 2 (PANK2) gene, neurodegeneration with brain iron accumulation (NBIA) is the most common, impacting 50% of cases [6]. These pantothenate kinase-associated neurodegenerative symptoms are connected to both the conventional and atypical PKAN symptoms. Classic PKAN is characterized by progressive stiffness, pigmentary retinal degeneration, dysarthria, dystonia, and choreoathetosis beginning in infancy (before six years of age in 88% of cases). Atypical PKAN is characterized by a later generation of onset (>10 years), apparent speech difficulties, psychosocial issues, and a slower pace of disease progression [7]. The PANK1a, PANK1b, PANK2, and PANK3 members of the pantothenate kinase gene family all contribute to PKAN, although only mutations in PANK2 do so. The PANK2 enzyme uses ATP to catalyze the conversion of (R)-pantothenate into (R)-4′-phosphopantothenate. Coenzyme A (CoA) thioesters and CoA provide feedback inhibition that carefully controls this process. The defective PANK2 enzyme hinders CoA biosynthesis, causing metabolic alterations, reduced energy production, and increased oxidative stress [8]. While considering additional signs of the patients or the course of their disease, the only cutoff point between classically and atypical PKAN is the age of six at the first sign of motor symptoms. As a result, the diagnosis does not accurately depict how the disease has progressed [9].

## Review

Search methodology

**Figure 1:**
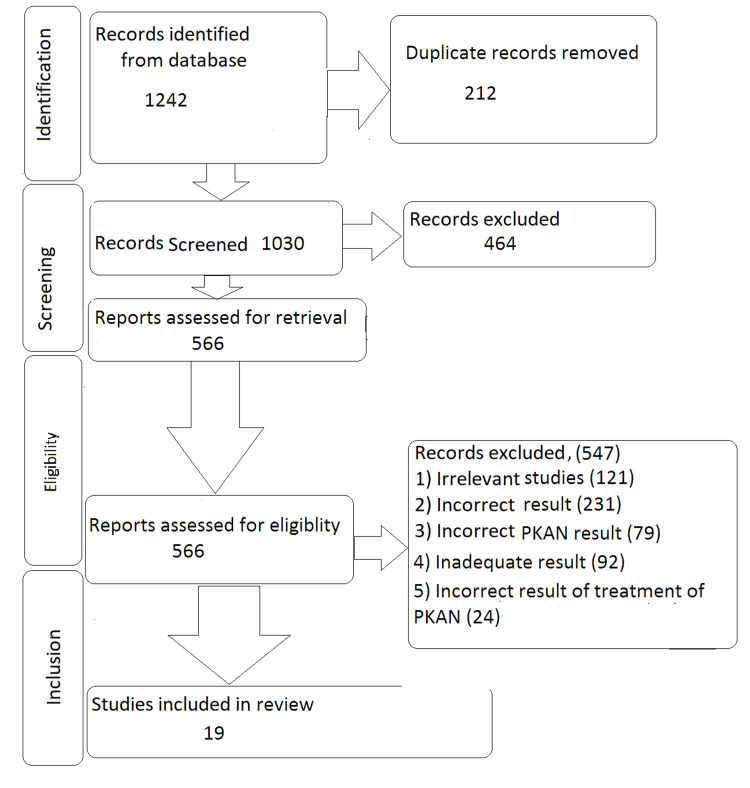
PRISMA flow chart of search strategy Adopted from the Preferred Reporting Items for Systematic Reviews and Meta-Analyses (PRISMA).

We undertook a systematic search through PubMed, Google Scholar, and CENTRAL in June 2023 using keywords such as “pantothenate kinase-associated neurodegeneration," “Iron/lipofuscin in pantothenate kinase-related neurodegeneration," “treatment," "diagnosis of PKAN, "Management"(((pantothenate kinase-associated neurodegeneration [Title/Abstract]) OR ("pantothenate kinase-associated neurodegeneration"[MeSH Terms]), (treatment [Title/Abstract])) OR (treatment [MeSH Terms]), (("management"[Title/Abstract]) OR ("management”[MeSH Terms]) AND ("diagnosis of PKAN” [Title/Abstract]) OR ("Diagnosis of PKAN”[MeSH Terms]). Another reviewer reviewed approximately 20% of these studies to validate inclusion studies. The selection of the studies depended on the following inclusion criteria: (1) PKAN, (2) management of PKAN full-text articles, (3) systematic reviews, and (4) English language. The following were the exclusion criteria: (1) opinion articles, (2) animal studies, (3) surveys, (4) no empirical study, and (5) non-English language research (Figure 1).

Neurodegeneration generated by pantothenate kinase in cellular models

Cytoplasmic inclusion bodies and circulating lymphocytes with vacuoles, macrophages in bone marrow express ceroid-lipofuscin granules. Similar to neuronal ceroid lipofuscinosis storage condition, there are two non-specific cytological abnormalities in NBIA cells, a process started by iron. High levels of lipid peroxidation can lead to the accumulation of lipofuscin. The investigation of these cytological aberrations may shed light on the pathophysiology of the NBIA underlying its fundamental level [10]. Fibroblasts from PANK2 mutation patients display physiopathological features, including lipofuscin accumulation, iron accumulation, increased reactive oxygen species (ROS), and mitochondrial dysfunction [11].

Iron/lipofuscin in pantothenate kinase-related neurodegeneration

Iron can be involved in potentially harmful free radical processes, and its buildup can aid in damage of nucleic acids and cause lipid/protein oxidation [12]. When iron builds up, it can engage in potentially harmful free extreme reactions and help create the hydroxyl radical, which damages nucleic acids and causes lipid/protein oxidation. As a result, iron accumulation can be seen in black and white blood cells as well as other parts of the brain. However, nothing is known about the chemical nature of iron deposition or the subcellular place where it occurs. Several theories have been put forth to clarify the mechanics underlying iron buildup [13]. When iron levels are low, cytosolic aconitase changes its shape and binds iron-responsive components that control the stability and translation rate of the mRNAs for ferritin and transferrin receptors. As a result, transferrin receptor mRNA becomes more stable, and ferritin mRNA translation is hindered [14]. In PKAN d-astrocytes, Perls reaction revealed over 50% iron-positive cells, compared to just 1% to 2% in controls; this stain had a granular pattern that is typical of cells that are iron-overloaded. The proportion of Perls-positive cells consistently decreased when the d-astrocytes developed in the presence of 25 M CoA, similar to what happened with derived mainly of striatal-like medium spiny neurons (d-MSNs). Electron-dense granules, similar to the aggregates seen with Perls staining, were found in PKAN d-astrocytes after ultrastructural investigations. Moreover, this research supported the existence of altered mitochondria, which were larger, bloated, and had damaged cristae [1].

Diagnosis for PKAN

Sample Blood Collection

The Muscle Culture Tissue Collection received blood from PKAN victims and associated carriers. Plasma was drawn in Munich and transported overnight at 4°C to 8 °C to Vienna. A healthy blood donation was also included in each sample to help control for changes brought on by shipping and time. The effects of a (almost) total loss of PANK2 function in erythrocytes from PKAN patients were also examined using metabolomics. Significant differences between PKAN erythrocytes and carrier and healthy control erythrocytes were found when metabolite levels in erythrocyte cytosolic extracts were analyzed. At the same time, the metabolic variability of erythrocyte samples is known to be donor- and storage-dependent [15].

Electrophysiological Investigations

Infantile design is linked to certain electrical anomalies that can help with diagnosis. Electroencephalography (EEG) frequently reveals quick, high-amplitude activity. Atypical nicotinamide adenine dinucleotide (NAD) has not been associated with fast EEG rhythms. However, some kids may experience epileptiform EEG abnormalities. Electromyography (EMG) and nerve conduction studies (NCS) may reveal denervation in infantile neuroaxonal dystrophy (INAD) and distal axonal-type sensory neuropathy. The diminished amplitude of visual evoked potentials is either missing or delayed [16].

Neuroimaging Features

Radiological characteristics on brain Sagittal T1-weighted sequence showing significant cerebellar atrophy (white arrow), apparent level hypertrophy (yellow arrow), MRI, and abrupt splenium alignment in the corpus callosum. Yellow indicators on T2-weighted sequences suggest hypointensity in the substantia nigra and globus pallidus, respectively. White matter alterations that are symmetrical are visible on imaging and can be reported in PLAN. Cerebellar atrophy is a common characteristic of INAD and is frequently the first MRI finding. In addition, but not always, INAD patients exhibit cerebellar gliosis. There are also reports of secondary cerebellar atrophy-related posterior corpus callosum anomalies, such as vertical orientation, thickness, and elongation of the splenium. Age-related increases in the severity of brain iron accumulation inside the substantia nigra, dentate nuclei, and (medial) globus pallidus are also described. Clinical optic atrophy is indicated by reduced optic nerve and chiasm volume. There have also been reports of alterations to the cerebral white matter, including shrinkage and elevated signal on T2-weighted sequences. In INAD, a consistent characteristic known as apparent "claval hypertrophy" has just been discovered [17].

Treatments for PKAN

There is currently no effective treatment for PKAN patients, and the many medications on the market have no impact on the disease's progression. Patients with dystonia may have a severe disability as the condition worsens and spreads to various body areas. Anticholinergics, botulinum toxin, intravenous and oral clonidine, gabapentin, baclofen, pregabalin, benzodiazepines, tetrabenazine, and other antispasticity medications are frequently used to treat it, either alone or in combination [18]. Vitamin B5 (pantothenate) could be able to help PKAN patients by improving CoA levels and reversing mitochondrial dysfunction. This medication prevented several neurological symptoms in PKAN animal models fed a high-fat ketogenic diet. However, future clinical trial research should be carried out to look at its impacts [19,20].

Surgery

Deep brain stimulation surgery is currently utilized to treat PKAN disease. Deep brain stimulation can have positive effects soon after surgery, but because the disease is progressive, symptoms may return later and worsen the condition [21]. Deep brain stimulation has mostly replaced other surgical procedures like ablative pallidotomy and thalamotomy, while they can still be used in conjunction with deep brain stimulation. Intrathecal baclofen (ITB) pumps can treat the symptoms of severe dystonia, spasticity, and pain in PKAN patients [22]. According to Blair Ford et al. and Albright et al., intraventricular baclofen effectively manages severe secondary and hereditary dystonia with spasticity and discomfort [23,24].

Iron Chelation 

Deferoxamine, deferiprone, and deferasirox are currently used [25]. Nowadays, a lot of scholars are interested in deferiprone. It can cross the blood and brain barrier to get rid of iron and stop it from building up [26,27]. Deferiprone was used safely and without adverse effects in studies examining its role in NBIA patients. Variable outcomes were achieved in these investigations where deferiprone lowered iron load in clinical evaluations and brain imaging, necessitating more studies in the future [28,29].

Alternative Treatments

According to observations, pantothenate can boost PANK2 expression in patient-derived fibroblasts with specific mutations [6]. Recently, an allosteric PANK activator (PZ-2891) that can penetrate the blood-brain barrier was discovered. The lifespan and locomotor activity of a knockout mice model of brain CoA deficiency were increased by PZ-2891 therapy, which is interesting. Observations imply that pantothenate can increase PANK2 expression levels in patient-derived cells carrying particular mutations [30].

Therapeutic Strategies for PKAN

Regarding PKAN, there has yet to be a disease-modifying therapy that has received FDA approval. The currently accepted standard of care focuses on treating specific symptoms. However, there are a number of effective remedies that are being actively researched right now [31].

Metabolite Loading

In order to treat PKAN, phosphopanthenate (PP) levels in PKAN cells should be restored. Drugs in this class's first generation were concentrated on getting exogenous P-Pa into harmed cells. Making the medication orally accessible and able to cross the blood-brain barrier was the biggest problem. Prodrugs with enhanced membrane permeability have been developed and have shown some promise in cell models. One of them, Fosmetpantotenate, has progressed to late-stage clinical trials [32]. However, the medication failed to achieve important primary and secondary objectives in a crucial stage II clinical trial with 84 individuals with PKAN, and as a result, further development was stopped. A cyclic phosphopantetheine acid prodrug that is theoretically comparable to Fosmetpantotenate recently demonstrated encouraging outcomes in a human PANK2/neuroblastoma cell line, elevating CoA levels. The prodrug improves phosphopantothenic acid levels in cells and has excellent physicochemical characteristics. It is still unknown whether this prodrug will work in clinical studies or mouse models of PKAN. [33].

Activation of CoA Biosynthesis

Small-molecule human PANK3 activators are a different strategy being investigated for treating PKAN. This strategy intends to activate PANK3, an isoform of human PANK ordinarily present in the cytosol or nucleus, to ameliorate abnormalities caused by mutations in PANK2. PZ-2891 was shown to activate PANK3 by inhibiting an allosteric site required for feedback inhibition by acetyl-CoA in a proof-of-concept preclinical research in mice [34]. Contrary to metabolite supplementation and iron chelation, an activator-based strategy could reduce the toxicity brought on by too much cysteine in PANk2-deficient cells. Regarding the promise of this strategy, there are still several open questions. CoA levels may not be necessary because patients may express PANK2 enzymes with activity close to WT [35].

Therapeutic Implications and PANK4 Phosphatase Activity

Because of two mutations in the catalytic area, PANK4's function in PKAN is generally disregarded [36]. Recombinant PANK4 DUF89 domain-containing moiety has increased preference for oxidized P-PaSH and phosphatase activity toward P-Pa, which may reduce CoA synthesis in PKAN patients and result in unpleasant symptoms [37].

Gene Therapies

There are prospects for gene therapy, including gene editing, in monogenic illnesses. To treat PKAN, a small functional copy of PANK2 can be inserted to make up for metabolic flaws. Functional proteins for Alzheimer's disease are carried via vectors [38]. Luxturna, a single-dose gene therapy for rare retinal degeneration, has received approval from the Food and Drug Administration [31]. Shortly, gene therapy for PKAN may be feasible, although difficulties with delivering vectors that must cross the blood-brain barrier exist. Adeno-associated vector advancements might solve this problem [39,40]. After a craniotomy, stereotactic infusion might offer gene therapies. However, getting reliable gene expression free of inflammation is still challenging. The long-lasting face provided by adeno-associated viruses carries little immunological danger [41]. Increased amounts of CoA in skeletal muscle were discovered in a study on transgenic mice overexpressing PANK2, along with reduced exercise tolerance and grip strength. Animal models should be used to assess the impact of gene therapy on physiology and metabolism [42].

Table 1 discusses the characteristics of the articles included in the review.

**Table 1:** Characteristics of the study included in the article

Author	Year	Country	Publications types	Findings
Santambrogio P, et al [1]	2022	Italy	Research Support, Non-U.S. Government	These PKAN astrocytes' examination revealed changes in iron metabolism, mitochondrial structure and function, respiratory activity, and oxidative state.
Hayflick SJ, et al [2]	2022	USA	Review	Considering the complexity of coenzyme A (CoA) metabolism and the limitations of our present understanding of disease causation, studies supporting several therapy methods for pantothenate kinase-associated neurodegeneration (PKAN) have been conducted.
Werning M, et al [9]	2022	Austria	Research Support, Non-U.S. Government	Findings point to the significance of carboxylate metabolism in PKAN disease, with possible connections to altered brain iron control and decreased cytoplasmic acetyl-CoA levels in neurons.
Munshi MI, et al [31]	2022	USA	Review	Gene treatments and PANK activators that activate CoA biosynthesis are two particularly promising strategies.
Tarnacka B, et al [5]	2021	Poland	Review	Researchers continue to disagree on whether chelator therapy is good or bad for those patients. During treatment, chelators also eliminate crucial trace metals.
Klopstock T, et al [32]	2021	Germany	Randomized Controlled Trial	In individuals with PKAN, fosmetpantotenate treatment was safe but did not enhance function as measured by the PKAN-Activities of Daily Living. Movement Disorders is a publication of the International Parkinson and Movement Disorder Society by Wiley Periodicals.
Auciello G, et al [33]	2020	Italy	Review	A recently discovered category of prodrugs has the ability to deliver phosphopantothenic acid (PPA) to the brain after oral treatment, and a proof-of-concept investigation in mice reveals incorporation of the prodrug-derived PPA into CoA.
Masud AJ, et al [3]	2019	Finland	Review	Information is mounting that mitochondrial acetyl-coenzyme A (CoA) levels are regulated by mitochondrial respiration, Fe-S cluster formation, and protein lipoylation, and that Acyl carrier protein (ACPs) produced by mitochondrial fatty acid synthesis (mtFAS) serve as signaling molecules in this circuit.
Marshall RD, et al [4]	2019	USA	Research Support, Non-U.S. Government	The frequent delays in PKAN diagnosis were most likely brought on by inadequate awareness. Across the PKAN severity continuum, significant functional impairment and excessive healthcare utilization were seen.
Alvarez-Cordoba M, et al [7]	2019	Spain	Review	According to PKAN, precision medicine entails creating medicines and treatment plans that are customized for each patient while taking both genetic and environmental factors into account.
Yao J, et al [36]	2019	USA	Research Support N.I.H. Extramural	This finding shows that prior to mutation of the catalytic residues throughout evolution, epistatic alterations to the remainder of the protein already decreased the kinase activity.
Di Meo I, et al [11]	2018	Italy	Review	Studies conducted in vitro and in vivo suggested that iron deposition may be a result of significant metabolic impairment in brain cells. This metabolic deficit appears to be brought on by various, though connected mechanisms.
Sharma LK, et al [34]	2018	USA	Research Support, N.I.H., Extramural	To develop PZ-2891, an allosteric PANK activator that penetrates the blood-brain barrier, we first did a library screen. Next, we optimized the chemical formula.
Hare DJ, et al [12]	2016	Australia	Review	We examine the clinical consequences of this molecular characteristic in this active and quickly developing area of Parkinson's disease research and explain that neurodegeneration in the affected regions may be caused by the strong redox couple created by iron and dopamine itself.
Hay Mele B, et al [13]	2015	Italy	Research Support, Non-U.S. Government	Pharmacological chaperone therapy, a promising approach to the treatment of genetic disorders, has recently been put forth. It makes use of inexpensive, orally administrable tiny molecules that can penetrate tough tissues like the brain.
Brunetti D, et al [20]	2012	Italy	Research Support, Non-U.S. Government	Demonstrate altered mitochondrial membrane potential in PANK2-faulty neurons generated, a defect that is further supported by the discovery of enlarged mitochondria at the ultra-structural level and by the presence of defective respiration.
Zhang YM, et al [35]	2006	USA	Research Support, N.I.H., Extramural	These findings characterized the distinct biochemical properties of PANK2 isoforms and provided evidence that catalytic deficiencies could not be the only factor causing the neurodegenerative phenotype.
Gregory A, et al [8]	2005	USA	Review	The identification of the gene, the clinical definition of PKAN, the development of animal models, and the investigation of potential therapies.
Perry TL, et al [10]	1985	Canada	Case Reports	The standard morphological alterations seen in this disorder may be caused by free radicals that harm neuronal membranes when cysteine and ferrous iron are present in excess.

## Conclusions

A mutation in the PANK2 gene causes PKAN, an autosomal recessive condition. Clinically, the disease can manifest as anything from a simple speech disorder to severe widespread spasticity, dementia, visual loss, dysphagia, dystonia, and dysphagia. Surgery, iron chelation, metabolite loading, COA biosynthesis activation, gene therapy, therapeutic implications, and PANK4 phosphatase activity were significant among the various treatment approaches in the PKAN disease. While the fact that some patient-derived cell lines' CoA levels appear to be the same as controls, several PKAN-related abnormalities are likely brought on by lower amounts of CoA or its downstream metabolites in the brain. The development of new therapies for this illness is currently in full swing. Gene treatments and the induction of CoA production by PANK activators are two promising strategies. PKAN precision medicine means developing treatments and therapies that work best for an individual patient, taking into account both genetic and environmental factors. It can be concluded that a personal approach to the treatment of PKAN will become more widely applicable in the future.
